# Prediction of leprosy in the Chinese population based on a weighted genetic risk score

**DOI:** 10.1371/journal.pntd.0006789

**Published:** 2018-09-19

**Authors:** Na Wang, Zhenzhen Wang, Chuan Wang, Xi'an Fu, Gongqi Yu, Zhenhua Yue, Tingting Liu, Huimin Zhang, Lulu Li, Mingfei Chen, Honglei Wang, Guiye Niu, Dan Liu, Mingkai Zhang, Yuanyuan Xu, Yan Zhang, Jinghui Li, Zhen Li, Jiabao You, Tongsheng Chu, Furong Li, Dianchang Liu, Hong Liu, Furen Zhang

**Affiliations:** 1 Shandong Provincial Hospital for Skin Diseases, Shandong University, Jinan, Shandong, China; 2 Shandong Provincial Institute of Dermatology and Venereology, Shandong Academy of Medical Sciences, Jinan, Shandong, China; 3 Shandong Provincial Key Lab for Dermatovenereology, Jinan, Shandong, China; 4 Shandong Provincial Medical Center for Dermatovenereology, Jinan, Shandong, China; University of California Davis, UNITED STATES

## Abstract

Genome wide association studies (GWASs) have revealed multiple genetic variants associated with leprosy in the Chinese population. The aim of our study was to utilize the genetic variants to construct a risk prediction model through a weighted genetic risk score (GRS) in a Chinese set and to further assess the performance of the model in identifying higher-risk contact individuals in an independent set. The highest prediction accuracy, with an area under the curve (AUC) of 0.743 (95% confidence interval (CI): 0.729–0.757), was achieved with a GRS encompassing 25 GWAS variants in a discovery set that included 2,144 people affected by leprosy and 2,671 controls. Individuals in the high-risk group, based on genetic factors (GRS > 28.06), have a 24.65 higher odds ratio (OR) for developing leprosy relative to those in the low-risk group (GRS≤18.17). The model was then applied to a validation set consisting of 1,385 people affected by leprosy and 7,541 individuals in contact with leprosy, which yielded a discriminatory ability with an AUC of 0.707 (95% CI: 0.691–0.723). When a GRS cut-off value of 22.38 was selected with the optimal sensitivity and specificity, it was found that 39.31% of high risk contact individuals should be screened in order to detect leprosy in 64.9% of those people affected by leprosy. In summary, we developed and validated a risk model for the prediction of leprosy that showed good discrimination capabilities, which may help physicians in the identification of patients coming into contact with leprosy and are at a higher-risk of developing this condition.

## Author summary

Despite elimination efforts, the reported number of new leprosy patients has been relatively stable during the past decade throughout the world. Solid evidence exists that individuals living in close proximity to patients are at an increased risk of developing leprosy, thus identifying the contact individuals who are at a higher risk of developing leprosy is an important aspect of disease control. In the last decade, genome-wide association studies (GWASs) have identified multiple genetic variants associated with leprosy in the Chinese population, however, the combined impact of these variants for leprosy risk prediction remains unclear. The goal of our study was to utilize the genetic variants to construct a risk prediction model in a Chinese set, and further assess the performance of the model in identifying higher-risk contact subjects in an independent set. We developed risk prediction models for leprosy based on GRS encompassing 25 GWAS-derived variants with good discriminatory capability (AUC = 0.743). When compared to the individuals in the high-risk group (GRS > 28.06) and low-risk group (GRS≤18.17), the former had a 24.65 times higher risk for developing leprosy than the latter, which demonstrated a considerable value for risk stratification in leprosy. Our results may assist physicians to identify higher-risk leprosy contact subjects for disease interventions.

## Introduction

Leprosy is a chronic granulomatous disease caused by *Mycobacterium leprae* that mainly affects the skin and peripheral nerves, potentially leading to irreversible disabilities and deformities. As a result of implementing multi-drug therapy, the prevalence of leprosy has declined dramatically. Nevertheless, the reported number of new leprosy patients (more than 200,000 new patients annually) has been relatively stable during the past decade globally [1, 2]. Endemic pockets remain in various parts of the world, especially in developing countries.

The development of leprosy in a non-leprous individual is highly dependent on the intensity of contact with a leprous patients [3, 4]. For decades, therefore, contact surveillance has always been a priority for disease control. In some endemic regions, post-exposure prophylaxis has been administered to prevent leprosy contacts (unaffected individuals coming into contact with leprosy) from contracting the disease. This has partially interrupted the transmission of the disease and reduced the incidence of leprosy [5–8]. However, it is noteworthy that most individuals exposed to this bacterium (95%) are not susceptible to leprosy, and amongst those 5% infected by *M*. *leprae*, only 1% go on to develop this condition [9]. Thus, the cost-effectiveness of chemoprophylaxis remains questionable and has not yet been widely approved. Use of a reliable risk prediction model that could inform clinicians of leprosy contact individuals at a higher risk of developing leprosy would allow the implementation of a more efficient strategy for disease interventions.

The discovery of susceptibility variants for human complex traits through genome-wide association studies (GWASs) has facilitated the potential application of genetic risk models, which could guide clinical professionals in their decision making by estimating an individual’s probability of having a special disease [10–13]. For leprosy, it has become increasingly apparent that, besides exposure to *M*. *leprae*, the host's genetic predisposition plays a critical role in the pathogenesis of the disease. There are currently 32 independent variants associated with leprosy that have been identified through GWAS and candidate-gene studies in the Chinese population [14–20]. Taking advantage of these findings, we utilized these published genetic risk variants to construct a risk prediction model using weighted genetic risk score (GRS) in a Chinese set. We then evaluated the risk model with respect to its discriminatory ability and found that it could achieve a substantial separation between people affected by leprosy and control individuals. To further assess the performance of the optimal risk model, we applied it to another independent set consisting of people affected by leprosy and leprosy contact individuals and demonstrated the effect of the model in identifying higher-risk contact individuals.

## Materials and methods

### Ethics statement

The study was approved by the institutional review board committee of the Shandong Provincial Institute of Dermatology and Venereology, Shandong Academy of Medical Science, China. We followed the Genetic Risk Prediction Studies guidelines [21] and all adult subjects provided written informed consent. A parent or guardian provided written informed consent on behalf of children who participated in the study.

### Study subjects

Two independent sets were enrolled in this study. The discovery set included 3,264 people affected by leprosy and 3,814 control subjects enrolled from 2006 to 2016. Although information regarding exposure to *M*. *leprae* remains unknown, the control subjects in this set were healthy individuals who had neither been diagnosed with nor had a family history of leprosy. The validation set consisted of 2,021 people affected by leprosy and 10,449 contact individuals recruited in the period from 2014 to 2016. Contact subjects were healthy individuals, who were categorized according to their genetic and physical distance to the index patient which included 5,983 relatives (first-, second-, and third- degree family members were 2,694, 2,218, and 1,071, respectively) and 4,466 genetically unrelated contact individuals (2,726 spouses and 1,740 neighbors). Generally, per index subject affected by leprosy, five individuals with prolonged, intimate contact were recruited. All self-reported Han Chinese subjects were from the Shandong Province in Northern China. The method associated with the diagnosis of leprosy has been previously described [14].

### SNP selection, genotyping and quality control

A total of 30 independent variants with minor allele frequencies > 0.01 at a genome-wide significance level were selected from our previous GWASs and one candidate gene study (**S1 Table**). The genotyping data from 1,572 patients and 2,484 control subjects in the discovery set was derived from our published GWASs database. The remaining subjects in the discovery set (1,692 people affected by leprosy and 1,330 controls) and all subjects in the validation set (2,021 people affected by leprosy and 10,449 contacts) were genotyped according to the manufactures’ protocol (dx.doi.org/10.17504/protocols.io.pvbdn2n) using the Quant Studio 12K Flex platform (Life Technologies, ABI, USA).

Variants went through the following quality control filters: call rate > 97% per variant and Hardy-Weinberg Equilibrium P > 1.0×10^−3^ in controls. Five variants with ≥ 3% missing data were eliminated. Subjects with missing data on one or more genetic variants of interest were also excluded from the analysis. Ultimately, a total of 25 variants and 13,741 subjects were included in the analyses.

### Statistical analysis

In the discovery set, we tested associations between phenotypes and single-variant genotypes using PLINK v 1.07 based on a logistic regression model. A two-sample t-test and Pearson χ^2^ test were conducted to compare the difference in age and gender between people affected by leprosy and controls, respectively.

Two risk prediction models were constructed using GRS in the discovery set. Both models were constructed as the sum of the risk alleles weighted by the β coefficient of each allele from a multivariate logistic regression of genetic covariates (weighted GRS). Model 1 included all genetic risk variants with a P value < 0.05, while only the top variants whose P values reached genome-wide significance (P < 5.0 × 10^−8^) in the discovery set were used to create GRS in model 2. This was because model 2 aimed to investigate the effectiveness of the simplified model. The Hosmer-Lemeshow test was used to evaluate for goodness of fit for the logistic regression models.

Receiver-operating characteristic (ROC) curves were applied to assess the discriminatory ability of the risk models. The area under the curve (AUC) and the 95% confidence intervals (CI) were calculated for each model. DeLong’s test from the pROC R package was used to test for statistically significant differences in AUCs obtained from different models [22].

To further assess the performance of the model, the probability (risk) cut-offs, sensitivity, specificity, and the number of subject needed for screening to prevent one case of leprosy were calculated in the discovery set. A positive likelihood ratio (PLR) above 5 was defined as having moderate evidence for leprosy, whereas a negative likelihood ratio (NLR) below 0.2 was considered to provide moderate evidence to exclude leprosy [23]. GRS cut-off values were selected based on the optimal PLR, NLR and the maximum sensitivity and specificity. To evaluate the risk between individuals in our study, subjects were divided into three risk groups according to optimal PLR and NLR at corresponding GRS cut-off values. Those with a predicted risk higher than that given by a cut-off value were defined as high-risk individuals.

### Accession numbers

***RIPK2*:** Gene ID: 8767. ***TNFSF15***: Gene ID:9966. ***LACC1***: Gene ID:144811. ***NOD2***: Gene ID:64127. ***HLA-DRB1***: Gene ID:3123. ***IL23R***: Gene ID: 149233. ***IL12B***: Gene ID:3593. ***CCDC122***: Gene ID:160857.

## Results

### Study subjects

After excluding subjects with any missing data, 2,144 people affected by leprosy and 2,671 controls were analyzed in the discovery set, while 1,385 people affected by leprosy and 7,541 contact individuals, which included 4,383 relatives (1,973 first-, 1,621 second-, 789 third-, degree family members) and 3,158 unrelated contact individuals (2,031 spouse and 1,127 neighbors), were finally used in the validation set. The baseline characteristics of these subjects are summarized in **Table 1**. Since no statistically significant differences were observed for age or gender between the people affected by leprosy and controls in the discovery set (P > 0.05), these two parameters were not included in further model construction.

**Table 1:** Characteristics GRS of study participants in discovery and validation sets.

	Discovery set		Validation set				
	People affected by leprosy (n = 2144)	Controls (n = 2671)	People affected by leprosy (n = 1385)	contacts (n = 7541)			
				First degree family members (n = 1973)	Second degree family members (n = 1621)	Third degree family members (n = 789)	Non-heredity-related contacts (n = 3158)
Age in years (mean ± SE)	66.87±8.42	63.07±9.98	72.52±9.28	55.33±14.6	37.57±19.14	46.23±22.22	58.52±14.29
Male sex (%)	81.2	79.1	78.05	66.85	64.16	71.61	32.71
GRS of 25 variants (mean ± SE)	23.94 ± 3.57	20.67± 3.59	23.70±3.58	22.03±3.62	21.62±3.59	21.15±3.68	20.92±3.67
OR(95% CI)*	NA	1.29 (1.27,1.32)	NA	1.14 (1.11,1.16)	1.18 (1.15,1.20)	1.22 (1.18,1.25)	1.23 (1.21,1.25)
P value*	NA	1.01E-152	NA	5.44E-36	1.52E-48	1.47E-45	2.59E-98

GRS, weighted genetic risk score

NA, not applicable

*OR and P values were from the comparison between people affected by leprosy and controls/contact individuals in the discovery and validation sets, respectively

### Association analysis

A total of 25 variants were successfully genotyped in the discovery and validation sets. All variants showed an association at P < 0.05 while the genetic risk effects between the current discovery set and previous GWASs were in concordance with one another. Seven of the 25 variants reached genome-wide significance in the discovery set. These included rs42490 at the *RIPK2* locus (P = 2.33 × 10^−13^), rs6478109 at the *TNFSF15* locus (P = 1.73 × 10^−11^), rs7995004 at the *LACC1* locus (P = 7.15 × 10^−28^), rs9302752 at the *NOD2* locus (P = 2.79 × 10^−37^), rs3762318 at the *IL23R* locus (P = 1.90 × 10^−16^), rs6871626 at the *IL12B* locus (P = 1.01 × 10^−10^) and rs9271100 at the *HLA-DRB1* locus (P = 6.32 × 10^−52^). The characteristics and association results of 25 variants are displayed in **S1 Table**.

### Construction and evaluation of the genetic risk model

**Fig 1:**
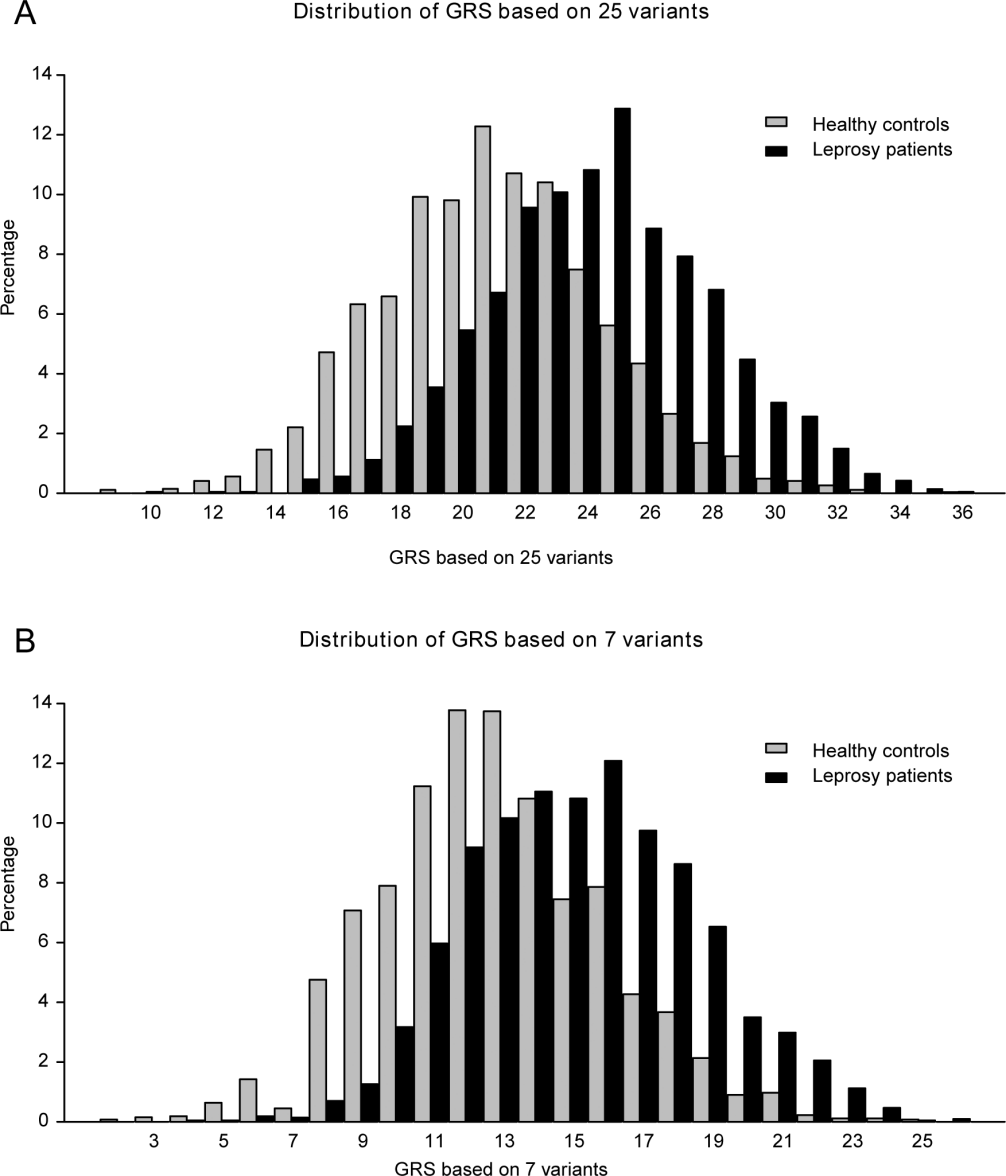
GRS Distribution. Distributions of weighted risk allele score (GRS) by leprosy–control status for model 1 (A) and model 2 (B).

We constructed two GRS prediction models using either all 25 variants (model 1) or the seven GWAS-significant variants (model 2) and compared their performance in predicting leprosy. Both models showed good fit following Hosmer-Lemeshow test evaluation (P > 0.05). The GRS distribution of leprosy-control status for model 1 and 2 is displayed in **Fig 1**. The GRS values in people affected by leprosy and controls showed an approximately normal distribution, but the people affected by leprosy tended to have a higher weighted risk score than controls.

**Fig 2:**
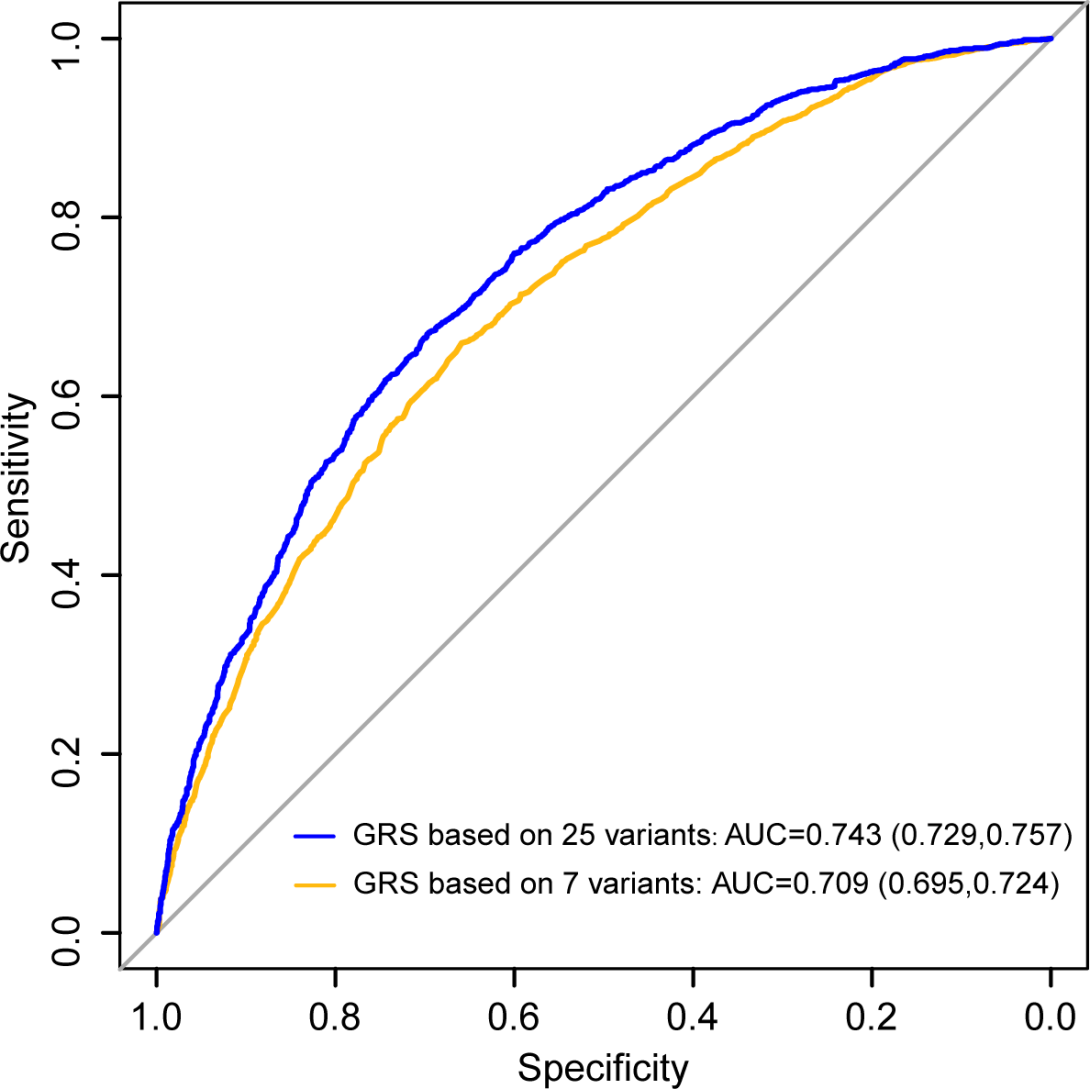
ROCs of the prediction models. ROC comparing model 1 with model 2.

The median GRS value of model 1 in the people affected by leprosy was 23.94 ± 3.57 and 20.67±3.59 in the controls, which was significantly different in favor of the people affected by leprosy (P = 1.01× 10^−152^, odds ratio (OR) = 1.29, 95% CI: 1.27–1.32, **Table 1**). In model 2, the median GRS values of the people affected by leprosy and controls were 14.86±3.26 and 12.32±3.25, respectively (P = 1.66 × 10^−122^, OR = 1.27, 95% CI: 1.24–1.29). The ability of the two models to discriminate between the leprosy and control individuals was compared by calculating the AUC. The AUC of model 1 was 0.743 (95% CI: 0.729–0.757) and 0.709 (95% CI: 0.695–0.724) for model 2 (**Fig 2**), and the change between these two models is 0.034 (95% CI: 0.026–0.042). Model 1 performed significantly better than model 2 in predicting the risk of developing leprosy (P = 2.12 × 10^−15^).

We set three GRS cut-off values according to the optimal PLRs and NLRs (PLR = 5.0 and NLR = 0.2) and the maximum sensitivity and specificity (sensitivity 67.1%, specificity 69.70%, PLR = 2.21). The corresponding sensitivity, specificity, positive predictive value (PPV), negative predictive value (NPV) and number of subjects needed to screen to prevent one case of leprosy of the three cut-off values are listed in **Table 2**.

**Table 2:** Genetic risk profile based on GRS in the model encompassing 25 variants.

GRS cut-off	Sensitivity	Specificity	PLR	NLR	PPV	NPV	NNT	"high risk" individuals number in validation set (rate)					
								People affected by leprosy (n = 1,385)	All contacts (n = 7,541)	First degree family members (n = 1,973)	Second degree family members (n = 1,621)	Third degree family members (n = 789)	Non-heredity-related contacts (n = 3,158)
18.17	95.10%	24.20%	1.25	0.20	6.34%	98.92%	21	1,309 (94.51%)	6,078 (80.60%)	1,682 (85.25%)	1,349 (83.22%)	617 (78.20%)	2,430 (76.95%)
22.38^&^	67.10%	69.70%	2.21	0.47	10.67%	97.52%	29	899 (64.9%)	2,964 (39.31%)	920 (46.6%)	685 (42.3%)	316 (40.1%)	1,043 (33.03%)
28.06	12.50%	97.50%	5.00	0.90	21.25%	95.38%	156	164 (11.84%)	291 (3.86%)	100 (5.07%)	60 (3.70%)	24 (3.04%)	107 (3.39%)

PLR, positive likelihood ratio; NLR, negative likelihood ratio

PPV, positive predictive value; NPV, negative predictive value

NNT, number needed to screen

^&^cut-off value corresponding to the maximum sensitivity and specificity

To evaluate the risk between subjects included in the discovery set, these individuals were divided into three groups (high-, intermediate- and low-risk). Two cut-off values (18.17 and 28.06), corresponding to a NLR of 0.2 and PLR of 5.0, were selected as the threshold for low- (4.94% of people affected by leprosy, 24.19% of controls) and high-risk groups (12.45% of people affected by leprosy, 2.47% of controls). Subjects with a GRS between 18.17 and 28.06 were treated as belonging to the intermediate group (82.60% of people affected by leprosy, 73.34% of controls). When comparing the high- and low-risk discovery groups to one another, the odds of developing leprosy was significantly higher in the subjects in the high-risk group than those individuals in the low risk group (OR = 24.65, 95% CI: 17.57–34.60; **Table 3**).

**Table 3:** Comparison of risk in different groups of individuals in discovery set.

Group	OR	95% CI	P
High vs. low risk	24.65	17.57–34.60	3.61E-99
High vs. intermediate risk	4.47	3.39–5.90	2.79E-30
Intermediate vs. low risk	5.51	4.44–6.83	2.80E-64

High-risk group: GRS > 28.06

Intermediate risk group: 18.17 < GRS ≤28.06

Low risk group: GRS ≤ 18.17

### The applicability of the genetic risk model in predicting leprosy

When applied to the validation set, model 1 displayed a discriminatory capability with an AUC of 0.707 (95% CI: 0.691–0.723) between people affected by leprosy and unrelated contact individuals. When comparing the median GRS values of people affected by leprosy to unrelated contact subjects, a significant difference was observed (P = 2.59 × 10^−98^, OR = 1.23, 95% CI: 1.21–1.25). The GRS of genetically related and unrelated contact individuals were significantly smaller than corresponding values observed in people affected by leprosy. The GRS value was also found to be inversely proportional to the genetic relationship of the contact individuals to those affected by leprosy (**Table 1**).

We further evaluated the effectiveness of the prediction by calculating the number of highrisk subjects above the cut-off point in the validation set and how much effort would be saved if the model could be used prior to tracing and performing prophylaxis on contact subjects (**Table 2**). With a cut-off of 18.17 and above, 94.51% of people affected by leprosy could be successfully identified with a sensitivity of 95.1% and a NPV of 98.92%. At the expense of a low specificity of 24.20%, 80.6% of the contact individuals would be classified as higher-risk contact subjects for preventive treatment. At a cut-off of 28.06 and above, only 11.84% of people affected by leprosy could be identified with a very limited sensitivity of 12.5%. At a higher specificity of 97.50% and a PPV of 21.25%, only 3.86% of contact subjects would be classified as higher-risk contact individuals for preventive treatment. At a cut-off of 22.38 and above, with the optimal sensitivity and specificity (67.1% and 69.7%, respectively), 64.9% of people affected by leprosy could be detected, while 39.31% of contact subjects should be screened.

## Discussion

By encompassing 25 variants in this study we developed a risk prediction model with good discriminatory capability for leprosy based on a GRS. The model of prediction performed better in the discovery set than in the validation set (AUCs = 0.743, and 0.707, respectively). This is likely due to the fact that some samples in the discovery set were from the original GWAS dataset, thus overestimating the performance due to over-fitting and the effect of winner’s curve. When compared to the individuals in the high-risk (GRS > 28.06) and low-risk groups (GRS ≤ 18.17), the former group had a 24.65 times higher risk for leprosy than the latter. This demonstrates the considerable value of risk stratification in leprosy. Furthermore, we displayed the clinical effect of this model in the identification of contacts at a higher risk of developing leprosy. Our findings highlight the potential of predicting disease risk from genetic variants associated with leprosy.

Over the past decade, with the ongoing advances in identifying genetic variants for complex diseases, genetic risk factors alone or in combination with clinical factors, have been widely implemented to establish the risk prediction models. There are some profound examples, especially in inflammatory/immune diseases and tumors, which have acquired highly variable levels of success in clinical practice. These include, but are not limited to, models for coeliac disease, age-related macular degeneration, breast cancer, and coronary heart disease [24–27]. Due to limited genetic findings, only a few risk models are available for the prediction of infectious diseases based on genetic variants and/or clinical factors. The predictive model for pulmonary tuberculosis, which incorporates six clinical factors along with ten genetic variants in a small set of 142 cases and 490 controls, has exhibited the highest AUC of 0.80 [28]. In community-acquired pneumonia and invasive aspergillosis models, AUC values did not reach 0.7 [29, 30]. Generally, a model with an AUC > 0.7 is considered useful in discriminating between high- and low-risk individuals. When it comes to the prediction of leprosy risk, *Zhang et al* (2016) was the first to report the contribution of the GRS derived from seven variants from our first GWAS dataset with an AUC of 0.701. This previously-reported model showed a similar discrimination capacity to model 2, as developed in this study, based on the top seven variants (AUC = 0.709). When combining all 25 identified variants, the predictive capability of our model was improved (AUC = 0.743). Although an increase of even 0.01 for the AUC might still be suggestive of a meaningful improvement [31], the modest improvement observed here indicates that variants beyond the seven included here have a limited contribution to disease risk.

As a millenary disease, interruption in the transmission of leprosy remains an important concern. Tracing and post-exposure chemoprophylaxis targeted at individuals coming into contact with leprosy has been carried out in some endemic countries with 35%–60% effectiveness being reported [5]. Nevertheless, given that only a small group of contact subjects (1%) will develop leprosy, attempts have been made to develop accurate risk profiles to narrow the population required for screening. This has included the detection of antibodies to the *M*. *leprae* phenolic glycolipid I (PGL-I) antigen among leprosy contact subjects. However, selection based on PGL-I testing has limited sensitivity (< 40%) and would miss more than half of the potential patients [32]. In Bangladesh, it was found that there is no association between anti-PGL-I Ab levels and the onset of disease [33], which further restricts the application of the PGL-I test. In terms of the potential clinical utility of the risk model constructed in this study, if the GRS cut-off value of 22.38 with optimal sensitivity and specificity is adopted, one case could theoretically be prevented by treating 29 contact individuals. In order to detect leprosy in 64.9% of the people affected by leprosy, 39.31% higher-risk contact subjects should receive preventive treatment, which appears to be cost-effective and easy to apply. Therefore, to some extent, the risk model can be used to identify contact individuals at a higher risk of developing leprosy in order to decrease the size of the population that should receive prophylactic treatment.

We acknowledge that there are several limitations to this study. Firstly, the genetic variants were all identified in the Chinese population. Only a few of these, such as *NOD2*, and *RIPK2* in Indian and Brazil, respectively [34, 35], and *RIPK2*, *CCDC122-LACC1*, and *NOD2* in Vietnam [36], have been identified in other ethnic populations. Thus, findings from our study may not be extended to other populations. Secondly, besides the genetic predisposition of the host, non-genetic factors such as the exposure to *M*. *leprae*, overcrowding, poor socioeconomic conditions, and gender have been reported to be important for the development of leprosy [37, 38]. These parameters were, however, not included in the present study due to the following reasons: 1) information regarding individual's exposure to the bacterium was missing; 2) all leprosy and control individuals were matched according to their region, socioeconomic status and environmental conditions in which they were living; and 3) no statistically significant differences was found between the gender groups for either leprosy or control subjects. Finally, the current study should be treated as a proof of concept to demonstrate that a genetic risk model could help to identify higher-risk contact individuals. Disease incidence statistics were not available for the contact subjects. As a result, we were unable to truly examine the discriminatory power of these variants for predicting the incidence of leprosy.

In conclusion, we have constructed a risk prediction model with good discrimination capacity using genetic variants associated with leprosy. This model may not only be used with reasonable confidence in identifying higher-risk contact subjects, but may also assist physicians in the control of leprosy by making decision to trace higher-risk contact individuals. However, the practical application of such risk stratification to clinical utility is yet to be evaluated. Further investigations should be done to determine the accuracy of the predictions in a prospective study.

## Supporting information

S1 Table The 25 variants associated with leprosy. (DOCX)
