# Supplementary material for: Prediction of leprosy in the Chinese population based on a weighted genetic risk score
Source: PLoS Negl Trop Dis. 2018 Sep 19;12(9):e0006789. doi: 10.1371/journal.pntd.0006789 (PMC6166985; doi:10.1371/journal.pntd.0006789)
Supplement: S1 Table — (DOCX) [file pntd.0006789.s001.docx]

| S1_Table: 25 variants associated with leprosy | | | | | | | | | | | | | | | | |  | | | | |  | | | | |  | | | |  | | | | | | | |  | | | | |  | | | |  | | | | | | | |  | | | | |  | | | | | |  | | | | | |  | | | | | | | |  | | | | | | | |
| --- | --- | --- | --- | --- | --- | --- | --- | --- | --- | --- | --- | --- | --- | --- | --- | --- | --- | --- | --- | --- | --- | --- | --- | --- | --- | --- | --- | --- | --- | --- | --- | --- | --- | --- | --- | --- | --- | --- | --- | --- | --- | --- | --- | --- | --- | --- | --- | --- | --- | --- | --- | --- | --- | --- | --- | --- | --- | --- | --- | --- | --- | --- | --- | --- | --- | --- | --- | --- | --- | --- | --- | --- | --- | --- | --- | --- | --- | --- | --- | --- | --- | --- | --- | --- | --- | --- | --- | --- |
| References | | | Reported SNP | | | | | | Gene | | | Gene ID | | | | | | Minor allele/  Major allele | | | | | | | Risk  allele | | | Pvalue^*^ | | | | | | OR^*^ | | | Minor allele frequency | | | | | | | | | | | | | | | | | | | | | | | | | | | | | | | | | | | | | | | | | | |  |  |  |  |  |  |  |  |  |
|  |  |  |  |  |  |  |  |  |  |  |  |  |  |  |  |  |  |  |  |  |  |  |  |  |  |  |  |  |  |  |  |  |  |  |  |  | Discovery cohort | | | | | | | | |  | | | | | | | | | Validation cohort | | | | | | | | | | | | | | | | | | | | | | | | | | | | | | | |  |  |
|  |  |  |  |  |  |  |  |  |  |  |  |  |  |  |  |  |  |  |  |  |  |  |  |  |  |  |  |  |  |  |  |  |  |  |  |  | Leprosy  patients (n=2144) | | | | | Controls (n=2671) | | | |  | | | Leprosy patients (n=1385) | | | | | | | | | leprosy patients' contacts (n=7541) | | | | | | | | | | | | | | | | | | | | | | | | |  |  |  |  |  |  |
|  |  |  |  |  |  |  |  |  |  |  |  |  |  |  |  |  |  |  |  |  |  |  |  |  |  |  |  |  |  |  |  |  |  |  |  |  |  |  |  |  |  |  |  |  |  |  | | |  | | | | | | | First degree family members (n=1973) | | | | | | | Second degree family members (n=1621) | | | | | | | | Third degree family members (n=789) | | | | | | | Non-heredity-related contacts(n=3158) | | | | | | | |  |  |  |
| NEJM^1^ | | | rs42490 | | | | | | *RIPK2* | | | 8767 | | | | | | A/G | | | | | | | G | | | 2.33E-13 | | | | | | 1.36 | | | 0.35 | | | | | 0.42 | | | |  | | | | 0.35 | | | | | | 0.38 | | | | | | | | 0.39 | | | | | | 0.41 | | | | | 0.42 | | | | | | | | |  |  |  |  |  |
| NEJM^1^ | | | rs6478109 | | | | | | *TNFSF15* | | | | | 9966 | | | | G/A | | | | | | | A | | | 1.73E-11 | | | | | | 1.32 | | | 0.45 | | | | | 0.52 | | | |  | | | | 0.44 | | | | | | | | | 0.48 | | | | | 0.50 | | | | | | 0.49 | | | | | 0.49 | | | | | | | | |  |  |  |  |  |
| NG^2^ | | | rs7995004 | | | | | | *LACC1* | | | 144811 | | | | | | T/C | | | | | | | T | | | 7.15E-28 | | | | | | 1.59 | | | 0.42 | | | | | 0.31 | | | |  | | | | 0.43 | | | | | | | | | 0.35 | | | | | 0.34 | | | | | | 0.34 | | | | | 0.32 | | | | | | | | |  |  |  |  |  |
| NEJM^1^ | | | rs9302752 | | | | | | *NOD2* | | | 64127 | | | | | | G/A | | | | | | | G | | | 2.79E-37 | | | | | | 1.72 | | | 0.43 | | | | | 0.30 | | | |  | | | | 0.42 | | | | | | | | | 0.36 | | | | | 0.34 | | | | | | 0.33 | | | | | 0.32 | | | | | | | | |  |  |  |  |  |
| NG^2^ | | | rs9271100 | | | | | *HLA-DRB1* | | | | | | 3123 | | | | T/C | | | | | | | T | | | 6.32E-52 | | | | | | 1.99 | | | 0.35 | | | | | 0.22 | | | |  | | | | 0.37 | | | | | | | | | 0.28 | | | | | 0.25 | | | | | | 0.21 | | | | | 0.21 | | | | | | | | |  |  |  |  |  |
| NG^3^ | | | rs3762318 | | | | | *IL23R* | | | | | | 149233 | | | | G/A | | | | | | | A | | | 1.90E-16 | | | | | | 1.80 | | | 0.07 | | | | | 0.12 | | | |  | | | | 0.07 | | | | | | | | | 0.09 | | | | | 0.09 | | | | | | 0.10 | | | | | 0.10 | | | | | | | | |  |  |  |  |  |
| NG^3^ | | | rs2275606 | | | | | | *RAB32* | | | 10981 | | | | | | A/G | | | | | | | A | | | 1.04E-06 | | | | | | 1.26 | | | 0.27 | | | | | 0.23 | | | |  | | | | 0.28 | | | | | | | | | 0.27 | | | | | 0.26 | | | | | | 0.26 | | | | | 0.25 | | | | | | | | |  |  |  |  |  |
| AJHG^4^ | rs2058660 | | | | | | *IL18RAP*  */IL18R1* | | | | | | | | | 8807/  8809 | | T/C | | | | | | | T | | | 2.35E-06 | | | | | | 1.21 | | | 0.50 | | | | | 0.45 | | | |  | | | | 0.48 | | | | | | | | | 0.45 | | | | | 0.44 | | | | | | 0.42 | | | | | 0.42 | | | | | | | | |  |  |  |  |  |
| AJHG^4^ | | | rs6871626 | | | | | | *IL12B* | | | 3593 | | | | | | A/C | | | | | | | C | | | 1.01E-10 | | | | | | 1.34 | | | 0.25 | | | | | 0.30 | | | |  | | | | 0.25 | | | | | | | | | 0.28 | | | | | 0.28 | | | | | | 0.30 | | | | | 0.29 | | | | | | | | |  |  |  |  |  |
| HMG^5^ | | | rs2735591 | | | | | | *BCL10* | | | 8915 | | | | | | T/C | | | | | | | T | | | 1.49E-03 | | | | | | 1.15 | | | 0.31 | | | | | 0.28 | | | |  | | | | 0.30 | | | | | | | | | 0.31 | | | | | 0.31 | | | | | | 0.30 | | | | | 0.29 | | | | | | | | |  |  |  |  |  |
| NG^2^ | | | rs2221593 | | | | | | *BATF3* | | | 55509 | | | | | | T/C | | | | | | | T | | | 1.28E-05 | | | | | | 1.24 | | | 0.23 | | | | | 0.20 | | | |  | | | | 0.22 | | | | | | | | | 0.22 | | | | | 0.21 | | | | | | 0.20 | | | | | 0.20 | | | | | | | | |  |  |  |  |  |
| NG^2^ | | | rs663743 | | | *CCDC88B* | | | | | | | | [283234](http://www.ncbi.nlm.nih.gov/entrez/query.fcgi?db=gene&cmd=Retrieve&dopt=full_report&list_uids=283234) | | | | A/G | | | | | | | A | | | 1.33E-05 | | | | | | 1.27 | | | 0.18 | | | | | 0.15 | | | |  | | | | 0.16 | | | | | | | | | 0.16 | | | | | 0.15 | | | | | | 0.15 | | | | | 0.16 | | | | | | | | |  |  |  |  |  |
| NG^2^ | | | rs77061563 | | | | | | *CIITA/*  *SOCS1* | | | | | | | 4261/  8651 | | T/C | | | | | | | C | | | 9.49E-04 | | | | | | 1.15 | | | 0.35 | | | | | 0.38 | | | |  | | | | 0.35 | | | | | | | | | 0.38 | | | | | 0.38 | | | | | | 0.38 | | | | | 0.39 | | | | | | | | |  |  |  |  |  |
| NG^2^ | | | rs160451 | | | | | | *RIPK2* | | | 8767 | | | | | | T/C | | | | | | | C | | | 2.30E-05 | | | | | | 1.24 | | | 0.19 | | | | | 0.23 | | | |  | | | | 0.21 | | | | | | | | | 0.23 | | | | | 0.22 | | | | | | 0.24 | | | | | 0.23 | | | | | | | | |  |  |  |  |  |
| NG^2^ | | | rs8002861 | | | | | | *LACC1* | | | 8767 | | | | | | G/A | | | | | | | A | | | 1.51E-02 | | | | | | 1.11 | | | 0.31 | | | | | 0.33 | | | |  | | | | 0.29 | | | | | | | | | 0.34 | | | | | 0.33 | | | | | | 0.34 | | | | | 0.35 | | | | | | | | |  |  |  |  |  |
| JID^6^ | | | rs76418789 | | | | | | *IL23R* | | | 8767 | | | | | | A/G | | | | | | | A | | | 1.16E-03 | | | | | | 1.33 | | | 0.07 | | | | | 0.05 | | | |  | | | | | | 0.07 | | | | | | 0.07 | | | | | | 0.07 | | | | | 0.06 | | | | | | | 0.06 | | | | | | | |  |  |  |  |  |
| JID^6^ | | | rs146466242 | | | | | | *FLG* | | | 2312 | | | | | | A/T | | | | | | | A | | | 2.03E-03 | | | | | | 1.33 | | | 0.06 | | | | | 0.04 | | | |  | | | | | | 0.04 | | | | | | 0.04 | | | | | | 0.05 | | | | | 0.05 | | | | | | | 0.05 | | | | | | | |  |  |  |  |  |
| JID^6^ | | | rs780668 | | | | | | *SLC29A3* | | | | | 55315 | | | | T/C | | | | | | | T | | | 4.13E-04 | | | | | | 1.16 | | | 0.47 | | | | | 0.43 | | | |  | | | | | | 0.47 | | | | | | 0.44 | | | | | | 0.44 | | | | | 0.46 | | | | | | | 0.43 | | | | | | | |  |  |  |  |  |
| JID^6^ | | | rs181206 | | | | | | *IL27* | | | | | 246778 | | | | G/A | | | | | | | A | | | 3.31E-03 | | | | | | 1.20 | | | 0.12 | | | | | 0.14 | | | |  | | | | | | 0.11 | | | | | | 0.13 | | | | | | 0.13 | | | | | 0.13 | | | | | | | 0.13 | | | | | | | |  |  |  |  |  |
| NC^7^ | | | rs6807915 | | | | | | *SYN2/PPARG* | | | | | | | 6854/  5468 | | C/T | | | | | | | T | | | 1.75E-03 | | | | | | 1.14 | | | 0.46 | | | | | 0.50 | | | |  | | | | | | 0.51 | | | | | | 0.50 | | | | | | 0.49 | | | | | 0.48 | | | | | | | 0.50 | | | | | | | |  |  |  |  |  |
| NC^7^ | | | rs55894533 | | | | | | *CTSB* | | | 1508 | | | | | | C/A | | | | | | | C | | | 2.59E-02 | | | | | | 1.10 | | | 0.42 | | | | | 0.40 | | | |  | | | | | | 0.43 | | | | | | 0.41 | | | | | | 0.41 | | | | | 0.41 | | | | | | | 0.40 | | | | | | | |  |  |  |  |  |
| NC^7^ | | | rs10100465 | | | | | | *MED30* | | | 90390 | | | | | | A/G | | | | | | | G | | | 5.56E-04 | | | | | | 1.17 | | | 0.26 | | | | | 0.29 | | | |  | | | | | | 0.28 | | | | | | 0.28 | | | | | | 0.28 | | | | | 0.28 | | | | | | | 0.28 | | | | | | | |  |  |  |  |  |
| unpublished | | | | rs13259978 | | | | | | *SLC7A2* | | | 6542 | | | | | | C/G | | | | | | | C | | | 5.71E-04 | | | | | | 1.28 | | | 0.10 | | | | | 0.08 | | | |  | | | | | | 0.10 | | | | | | 0.09 | | | | | | 0.10 | | | | | 0.11 | | | | | | | 0.10 | | | | | | | |  |  |  |  |
|  |  |  |  | rs671 | | | | | | *ALDH2* | | | 217 | | | | | | A/G | | | | | | | A | | | 7.69E-06 | | | | | | 1.27 | | | 0.20 | | | | | 0.16 | | | |  | | | | | | 0.20 | | | | | | 0.19 | | | | | | 0.18 | | | | | 0.17 | | | | | | | 0.18 | | | | | | | |  |  |  |  |
|  | | | | rs75680863 | | | | | | *TCN2* | | | 6948 | | | | | | T/A | | | | | | | A | | | 1.04E-03 | | | | | | 1.18 | | | 0.18 | | | | | 0.21 | | | |  | | | | | | 0.19 | | | | | | 0.19 | | | | | | 0.18 | | | | | 0.18 | | | | | | | 0.17 | | | | | | | |  |  |  |  |
|  | |  | | |  | | | | | |  | | | |  | | | | |  | | | |  | | | | | |  | | |  | | | | | | |  | | | | |  | | | | | |  | | | | | |  | | | | |  | | | | | |  | | | | | |  | | | | | | | |  |  |  |  |  |  |  |
| OR, odds ratio is with respect to the risk allele  *Association results from discovery cohort | | | | | | | | | | | | | | | | | | | | |  | |  | | | | | | | | |  | | | |  | | | | |  | | | | | | | | | | | | |  | | | | | |  | | | | | |  | | | | | |  | | | | | | |  | | | | | | | | |  |

**Reference**

1. Zhang FR, Huang W, Chen SM, Sun LD, Liu H, Li Y, et al. Genomewide association study of leprosy. N Engl J Med 2009;361:2609-18.

2. Liu H, Irwanto A, Fu X, Yu G, Yu Y, Sun Y, et al. Discovery of six new susceptibility loci and analysis of pleiotropic effects in leprosy. Nat Genet 2015;47:267-71.

3. Zhang F, Liu H, Chen S, Low H, Sun L, Cui Y, et al. Identification of two new loci at IL23R and RAB32 that influence susceptibility to leprosy. Nat Genet 2011;43:1247-51.

4. Liu H, Irwanto A, Tian H, Fu X, Yu Y, Yu G, et al. Identification of IL18RAP/IL18R1 and IL12B as leprosy risk genes demonstrates shared pathogenesis between inflammation and infectious diseases. Am J Hum Genet 2012;91:935-41.

5. Liu H, Bao F, Irwanto A, Fu X, Lu N, Yu G, et al. An association study of TOLL and CARD with leprosy susceptibility in Chinese population. Hum Mol Genet 2013;22:4430-7.

6. Liu H, Wang Z, Li Y, Yu G, Fu X, Wang C, et al. Genome-Wide Analysis of Protein-Coding Variants in Leprosy. The Journal of investigative dermatology 2017;137:2544-51.

7. Wang Z, Sun Y, Fu X, Yu G, Wang C, Bao F, et al. A large-scale genome-wide association and meta-analysis identified four novel susceptibility loci for leprosy. Nat Commun 2016;7:13760.
